# In Vivo and In Silico Investigation of the Anti-Obesity Effects of *Lactiplantibacillus plantarum* Combined with Chia Seeds, Green Tea, and Chitosan in Alleviating Hyperlipidemia and Inflammation

**DOI:** 10.3390/ijms232012200

**Published:** 2022-10-13

**Authors:** Dalia Elebeedy, Aml Ghanem, Asmaa Saleh, Mona H. Ibrahim, Omkulthom Al Kamaly, Mohammed A. S. Abourehab, Mohamed A. Ali, Ahmed I. Abd El Maksoud, Mahmoud A. El Hassab, Wagdy M. Eldehna

**Affiliations:** 1 Pharmaceutical Biotechnology Department, College of Biotechnology, Misr University for Science and Technology (MUST), 6th of October City 12573, Egypt; 2 School of Biotechnology, Badr University in Cairo, Cairo 11829, Egypt; 3 Department of Pharmaceutical Sciences, College of Pharmacy, Princess Nourah bint Abdulrahman University, P.O. Box 84428, Riyadh 11671, Saudi Arabia; 4 Department of Pharmaceutical Medicinal Chemistry and Drug Design, Faculty of Pharmacy for Girls, Al-Azhar University, Cairo 11754, Egypt; 5 Department of Pharmaceutics, Faculty of Pharmacy, Umm Al-Qura University, P.O. Box 21961, Makkah 24382, Saudi Arabia; 6 Industrial Biotechnology Department, Genetic Engineering and Biotechnology Research Institute, University of Sadat City, Sadat City 32897, Egypt; 7 Department of Medicinal Chemistry, Faculty of Pharmacy, King Salman International University (KSIU), South Sinai 46612, Egypt; 8 Department of Pharmaceutical Chemistry, Faculty of Pharmacy, Kafrelsheikh University, Kafrelsheikh 33511, Egypt

**Keywords:** *Lactiplantibacillus plantarum*, prebiotics, chia seeds, green tea, chitosan, obesity, nutrition, docking

## Abstract

The increasing prevalence of obesity has become a demanding issue in both high-income and low-income countries. Treating obesity is challenging as the treatment options have many limitations. Recently, diet modification has been commonly applied to control or prevent obesity and its risks. In this study, we investigated novel therapeutic approaches using a combination of a potential probiotic source with prebiotics. Forty-eight adult male Sprague–Dawley rats were selected and divided into seven groups (eight rats per group). The first group was fed a high-fat diet, while the second group was a negative control. The other five groups were orally administered with a probiotic, *Lactiplantibacillus plantarum* (*L. plantarum*), and potential prebiotics sources (chia seeds, green tea, and chitosan) either individually or in combination for 45 days. We collected blood samples to analyze the biochemical parameters and dissected organs, including the liver, kidney, and pancreas, to evaluate obesity-related injuries. We observed a more significant decrease in the total body weight by combining these approaches than with individual agents. Moreover, treating the obese rats with this combination decreased serum catalase, superoxide dismutase, and liver malondialdehyde levels. A histopathological examination revealed a reduction in obesity-related injuries in the liver, kidney, and pancreas. Further docking studies indicated the potential role of chia seeds and green tea components in modulating obesity and its related problems. Therefore, we suggest that the daily administration of a pre- and probiotic combination may reduce obesity and its related problems.

## 1. Introduction

Obesity is one of the major contributors to the global health crisis of metabolic disease, as [1] it occurs due to the abnormal accumulation of excessive fat within adipose tissues [2]; it causes many complications that often lead to several human health issues by predisposing the individual to diseases such as diabetes type II, atherosclerosis, hypertension, cardiovascular diseases, metabolic syndrome, and inflammation [3,4]. In 2014, the World Health Organization (WHO) claimed that more than 1.9 billion persons were overweight and more than 600 million were obese [5]. Overweight and obesity, which were previously primarily a concern for higher-income countries, are now also on the rise in low- and middle-income countries, especially in urban areas, as a result of a lack of awareness of the complications of obesity in these areas and the high cost of anti-obesity medications [6,7,8]. Body mass index (BMI) is a more advanced and standardized method to determine and classify obesity [9]. It is calculated as the weight (in kg)/height (in m^2^). According to the WHO, the prevalence of obesity (BMI ≥ 30 kg/m^2^) is expected to increase from 33% in 2005 to 57.8% in 2030 [10,11]. 

Obesity is linked with systemic oxidative stress resulting from the peroxisomal and mitochondrial oxidation of the adipose tissues fatty acids, leading to liver failure, inflammation, and damage [3,4]. Collectively, these events make obesity a global epidemic and a major challenge in the coming years [12,13]. Although several treatment approaches are available, they have serious limitations. When obese patients are treated with administrated drugs, they can consume an adequate basic diet with minimal side effects such as gastrointestinal discomfort, high blood pressure, constipation, headache, heart attack, and insomnia [14,15]. 

Therefore, natural diet modification is recommended to control or prevent obesity and its risks. First concepts for employing pre- and probiotics in combination as a natural therapy for the treatment of obesity have been established by Cava et al. [16]. Le Barz et al., and Krentz et al., who found that the presence of antioxidants makes *Lactiplantibacillus plantarum* (*L. plantarum*) a promising source for probiotics, which can protect the gut from inflammation by modulating the reactive oxygen species (ROS) [17,18,19,20]; they also have prophylactic abilities such as reducing free triglycerides and leptins [19,21], by using high-fat diet (HFD)-fed mice [22]. However, the underlying mechanism is still unclear. 

Prebiotics, which are found in different sources such as the seeds of *Salvia hispanica L.* or chia seeds, contain potentially bioactive peptides including angiotensin-converting enzyme inhibitors that have antioxidant and anticholesterolemic activity, making them a significant tool to manage and control obesity [23,24]. Green tea leaves are known for their hypolipidemic and anti-obesity effects, which may be related to their high polyphenols, alkaloids, volatile oils, vitamins, and/or amino acids content [25]. Moreover, they can efficiently decrease serum leptin levels [26,27]. The powder of chitosan (polysaccharide in the linear shape, randomly composed of distributed β-(1→4)-linked D-glucosamine (deacetylated unit) and N-acetyl-D-glucosamine (acetylated unit)), which is a key component of the exoskeleton of several insects, has been shown to have anti-obesity properties due to the presence of glucosamine and N-acetylglucosamine [28,29]. Chitosan can inhibit weight gain by improving dyslipidemia, accelerating lipid oxidative catabolism, and by acting as a carrier for cholesterol and triglycerides [30,31]. 

This study aims to evaluate the anti-obesity effects of *Lactobacillus plantarum* combined with chia seeds, green tea, and chitosan in alleviating hyper-lipidemia and inflammation.

## 2. Results and Discussion

The pathogenesis of obesity involves multiple factors such as a sedentary lifestyle and hormonal disturbance. Diet control and lifestyle modifications are the primary therapy for controlling obesity and related diseases. Our study revealed the effects of prebiotics and probiotics on the weight, lipid profile, and internal organ damage in HFD-fed rats. 

### 2.1. Effects of Prebiotic and Probiotic on the Weight of HFD Rats

**Figure 1:**
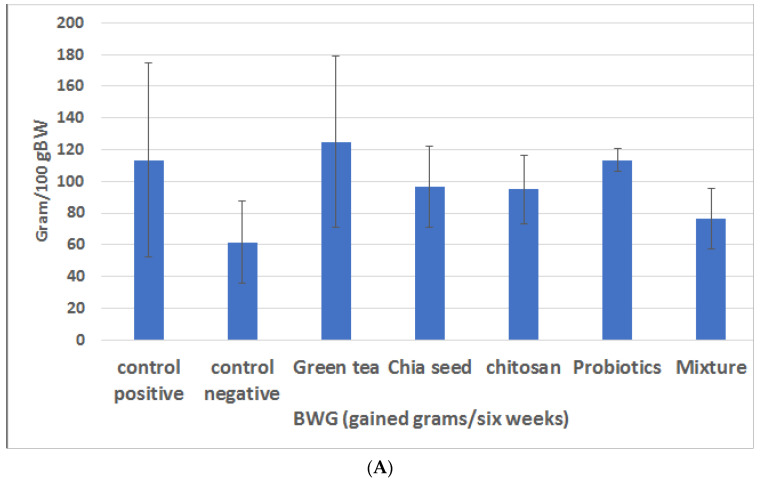

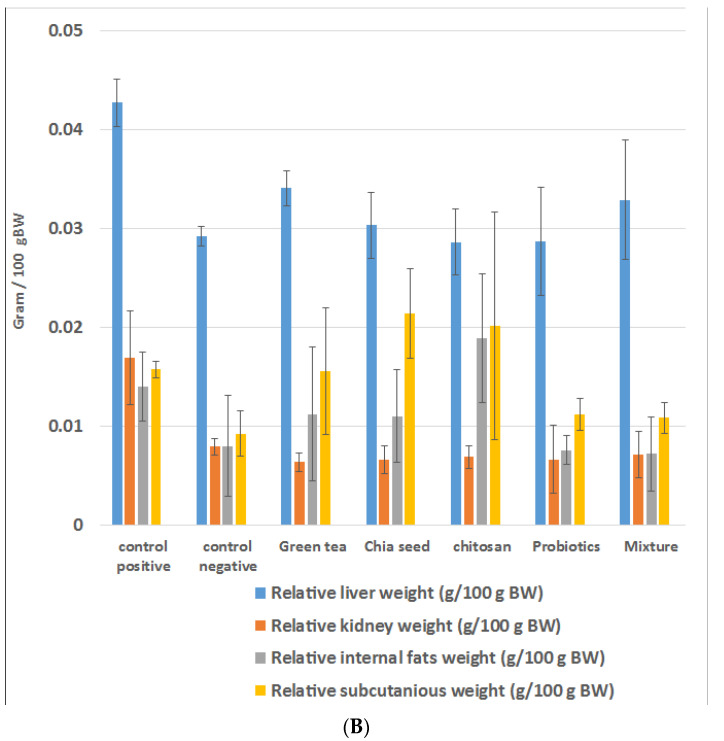
(**A**) Effect of prebiotics and probiotics on the weight of rats fed high-fat diet. (**B**) The body weight gain (BWG) at the end of experiment; the relative liver weights.

Probiotics are described as "live bacteria such as Lactobacillus that, when supplied in suitable proportions, impart a health benefit on the host" by the WHO and the Food and Agriculture Organization (FAO) of the United Nations. The administration of an adequate mixture of probiotics and prebiotics reduced the BW [32]. We measured the BWG, relative internal and subcutaneous fat content, and relative kidney and liver weights for all the experimental rats six weeks after administering the suitable weight-control plans. As shown in Figure 1A,B, the BWG of the HFD-fed rats was significantly higher when compared with that in the negative control group (113 g ± 14 g versus 62 g ± 6 g, at *p* < 0.05). After receiving a mixture of probiotic and prebiotic, the HFD-fed rats showed significantly lower BWG (77 g ± 4 g), relative liver weights (0.03288 g ± 0.001 g), relative kidney weights (0.0072 g ± 0.0005 g), and relative subcutaneous fat weight (0.010859 g ± 0.00 g) with *p* values of <0.05; see supplementary data for further information.

The individual administration of prebiotics and probiotics significantly decreased the relative liver and kidney weights, and the relative subcutaneous fat weight, with little effect on the total BWG. The administration of green tea extract did not affect the BWG but significantly decreased the internal organ weight, as shown in previous studies. Chen et al. showed that when green tea extract was added to HFD, it effectively reduced the animals’ adipose tissue mass [33]. Similar results, i.e., significant reduction in BWG and liver weight, were seen when chia seeds were fed to rats [34,35,36]. In our study, rats fed with HFD + chia seeds showed a significant decrease in both BWG and the relative organ weights. Chitosan is considered healthy and is usually well-absorbed with strong biocompatibility and biodegradability [37,38,39]. It can also effectively decrease the internal organs’ weights, as confirmed by some studies evaluating the modulatory effect of probiotics on BW and BMI [12,39,40,41]. 

Our study showed that probiotics could decrease the BWG and the weights of some organs, as previously reported [42]. Consequently, we aimed to study the effect of combining prebiotic and probiotic, using various feeding plans, on HFD-fed rats. Our results revealed that this combination was superior to the individual agents and could effectively reduce the BWG, organ weights, and subcutaneous fat of the HFD-fed rats compared with the controls. This is consistent with previous studies showing that prebiotics had a neutral effect on BW and probiotics had a small but significant effect on BW, but that their combination induced changes in the microbiota, resulting in higher weight reduction [43]. Therefore, we suggested that *L. plantarum* decreases fat accumulation and chronic inflammation along with changes in the microbiota composition, as well as combating oxidative stress [44,45].

### 2.2. Effects of Prebiotics and Probiotic on the Lipid Profile

**Figure 2:**
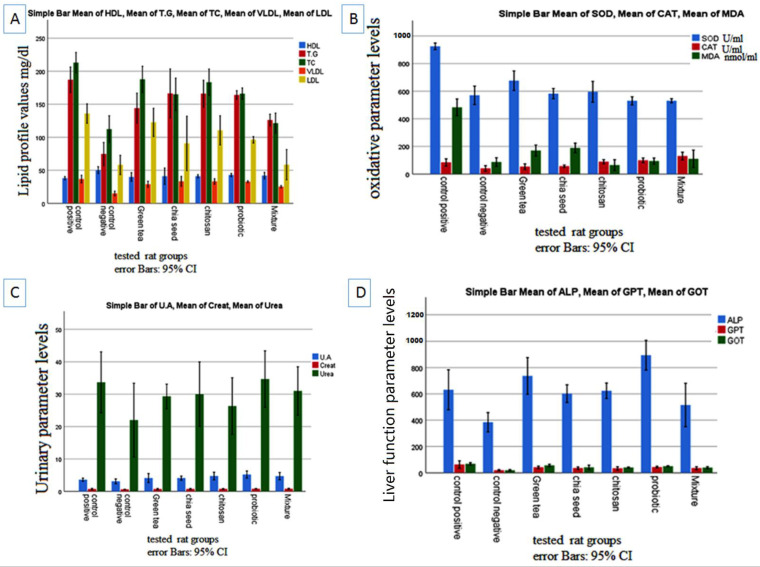
Effect of prebiotics and probiotics on rats fed with high-fat diet. (**A**) Lipid profile parameters; (**B**) oxidative parameter levels; (**C**) urinary parameter; (**D**) liver function.

It has been demonstrated that administering probiotics and prebiotics improves the lipid profile [46]. According to our study, the serum levels of triglyceride (TG), total cholesterol (TC), and low-density lipoprotein (LDL) showed a significant increase in the HFD group, indicating abnormal blood lipid metabolism. Conversely, the oral administration of lactobacilli markedly lowered the TC, TG, LDL, and very low-density lipoprotein (VLDL) values. Moreover, the individual administration of prebiotics and probiotics had little effect on these lipid parameters. However, we did not detect any significant change in HDL levels in any group upon investigating the oxidative stress and the effect of prebiotics and probiotics in the HFD group (Figure 2A).

As shown in Figure 2B, the HFD group displayed significant oxidative stress compared with the SBD group (*p* < 0.05). The administration of prebiotics, probiotics, or their mixture significantly lowered oxidative stress. The results showed significantly higher serum levels of UA, Cr, ALP, GPT, and GOT in the HFD group without any change in the serum urea level. When a mixture of prebiotic and probiotic was administered, we observed significantly lower levels of ALP (515 ± 38 versus 631 ± 35, at *p* < 0.05) and GOT (39 ± 2 versus 68 ± 2, *p* < 0.05) (Figure 2C,D); see supplementary data for further information. 

These results were consistent with a study by Chen et al., which found that green tea extract effectively improved plasma lipid profiles, relieved oxidative stress, and reduced elevated liver enzymes in HFD-fed rats [33]. In this study, we report a synergistic effect of prebiotics and probiotics in ameliorating oxidative stress and improving liver function. 

### 2.3. Effects of Prebiotics and Probiotics on Internal Organ Damage 

**Figure 3:**
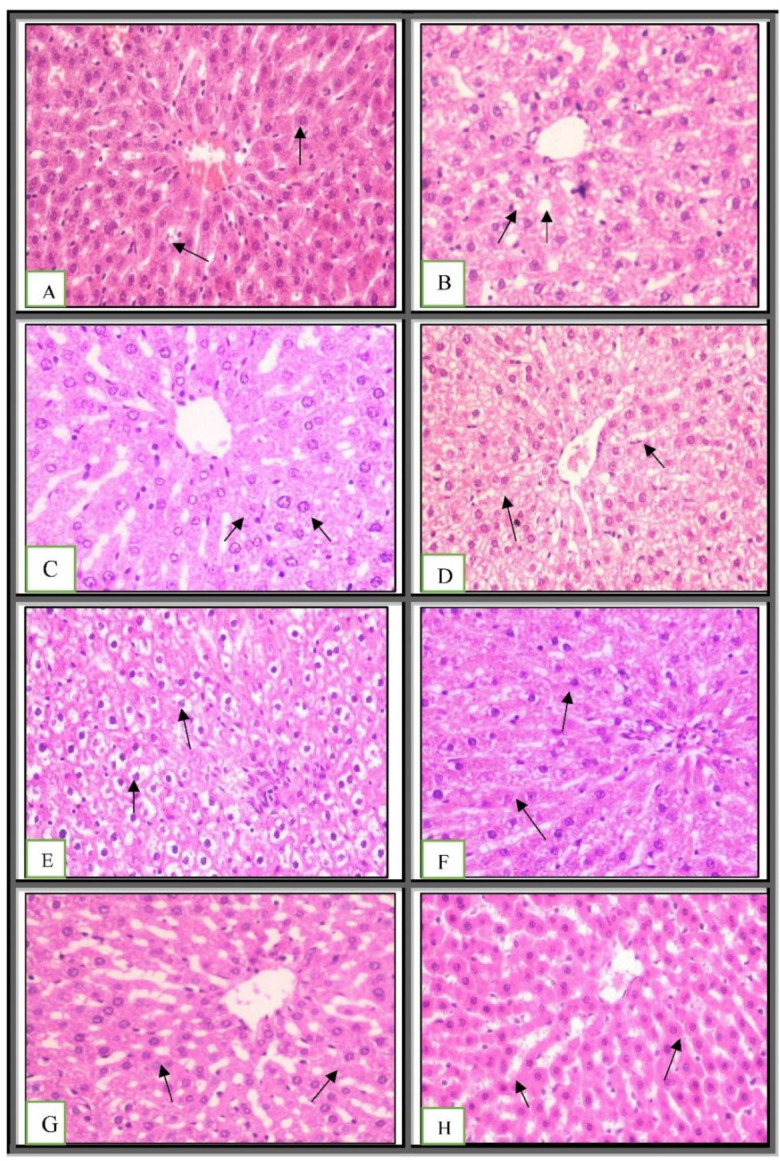
Histopathological examination of hepatic tissue: (**A**) showing normal histological structure of hepatic lobule **arrow**; (**B**) showing ballooning and fatty degeneration of hepatocytes **arrow** (H&E ×400); (**C**) showing mild swelling of hepatocytes **arrow**; (**D**) showing ballooning degeneration of hepatocytes **arrow** (H&E ×400); (**E**) showing ballooning degeneration of hepatocytes **arrow**; (**F**) showing swelling of hepatocytes **arrow** (H&E ×400); (**G**,**H**) showing no apparent injury by light microscopy **arrow** (H&E ×400). (**A**) Negative control; (**B**) positive control; (**C**,**D**) green tea; (**E**) chia seed; (**F**) chitosan; (**G**) probiotic; (**H**) mixture. A 50 µM bar scale was used for all the tissues.

The gut microbiota is improved by a prebiotic and probiotic-rich diet because prebiotics promote probiotic development [47]. Pre-probiotics are used in conjunction with other medications to treat obesity-related colitis and hepatic symptoms [48], skeletal health and immunity [49], metabolic profile in chronic kidney disease [50], and psychological outcomes in obese patients [51]. The control liver showed normal large polygonal cells with prominent round nuclei and eosinophilic cytoplasm, as well as a few spaced hepatic sinusoids arranged in between the hepatic cords and a fine arrangement of Kupffer cells, upon microscopic examination of the histopathological section of the liver (grade 0) (Figure 3A and Table 1). However, the HFD-fed animals showed a ballooning and fatty degeneration of hepatic cells, a narrowing of hepatic sinusoids, and hyperplasia of the Kupffer cells (grade III) (Figure 3B). The animals treated with green tea showed mild swelling in the hepatocytes and cytoplasmic granularity (grade I) (Figure 3C,D), while those treated with chia seed or chitosan showed similar histopathological features to those of the control group, except for the ballooning of the hepatocytes (grade II) (Figure 3E,F). These results were not consistent with that observed by Xin Zhao et al., who described the protective effect of *L. plantarum* against obesity-associated pathological changes in the livers of animals fed with probiotic feeding along with a lack of apparent injury, which was shown using light microscopy analysis (grade 0) (Figure 3G) [52]. However, the animals treated with a mixture of the selected prebiotic and probiotic showed similar histopathological features to those of the control group, which were characterized by a lack of apparent injury, as observed using light microscopy (grade 0) (Figure 3H).

**Figure 4:**
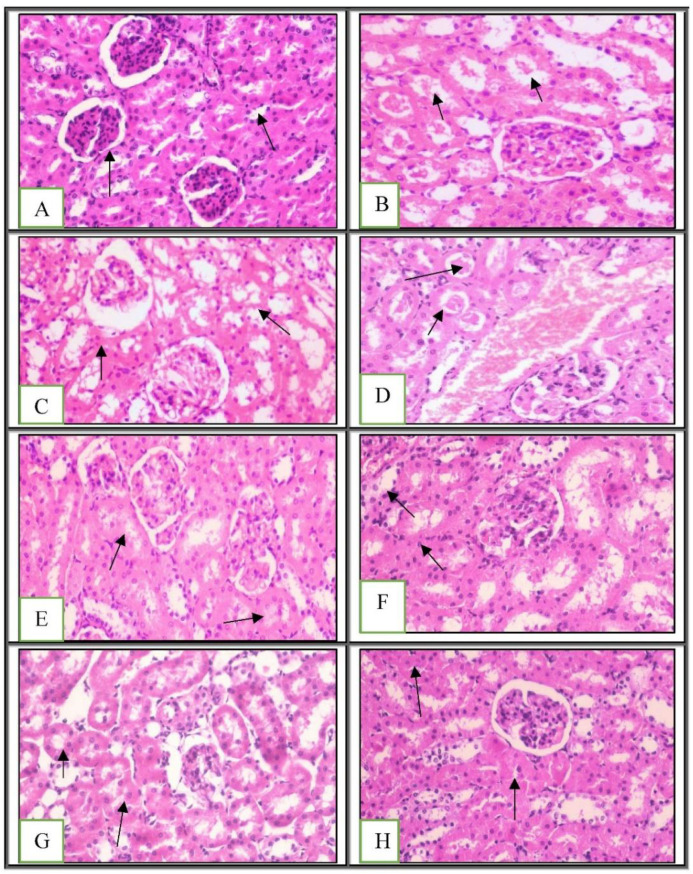
Histopathological examination of kidney tissues: (**A**) showing normal histological structure of kidney parenchyma **arrow score** (**0**) (H&E ×400); (**B–D**) showing tubular epithelial cell degeneration with intraluminal albuminous material **arrow score** (**2**) (H&E ×400); (**E**–**H**) showing tubular epithelial cell degeneration and narrowing of tubular lumen **arrow score** (**1**) (H&E ×400). (**A**) Negative control; (**B**) positive control; (**C**,**D**) green tea; (**E**) chia seed; (**F**) chitosan; (**G**) probiotic; (**H**) mixture. A 50 µM bar scale was used for all the tissues.

When examined under a microscope, the kidney sections from the experimental animals revealed a typical Bowman’s capsule architecture and circumscribing glomeruli with normal capillary tufts. Both the proximal and distal convoluted renal tubules displayed an intact epithelial lining and an arrangement scoring of zero (Figure 4A and Table 2). However, the kidneys of the positive control and the green tea-treated groups showed shrunken capillary tufts with a widening of Bowman’s space in some glomeruli. Epithelial cell degeneration and intraluminal albuminous material with considerable necrosis or apoptosis with a score of less than 25% were present in the renal tubules (Figure 4B–D). The kidneys of the rats treated individually with probiotics, chia seeds, chitosan, and their mixture showed a normal histological structure of the renal glomeruli. The epithelial cell degeneration in the renal tubules was characterized by swelling of the epithelial lining and tubular lumen constriction without obvious necrosis or apoptosis scoring (1) (Figure 4E–H). These findings are in line with other research suggesting that a synbiotic (a combination of prebiotics and probiotics) supplement may be able to prevent the progression of chronic kidney disease to end-stage renal disease (ESRD) [53].

**Figure 5:**
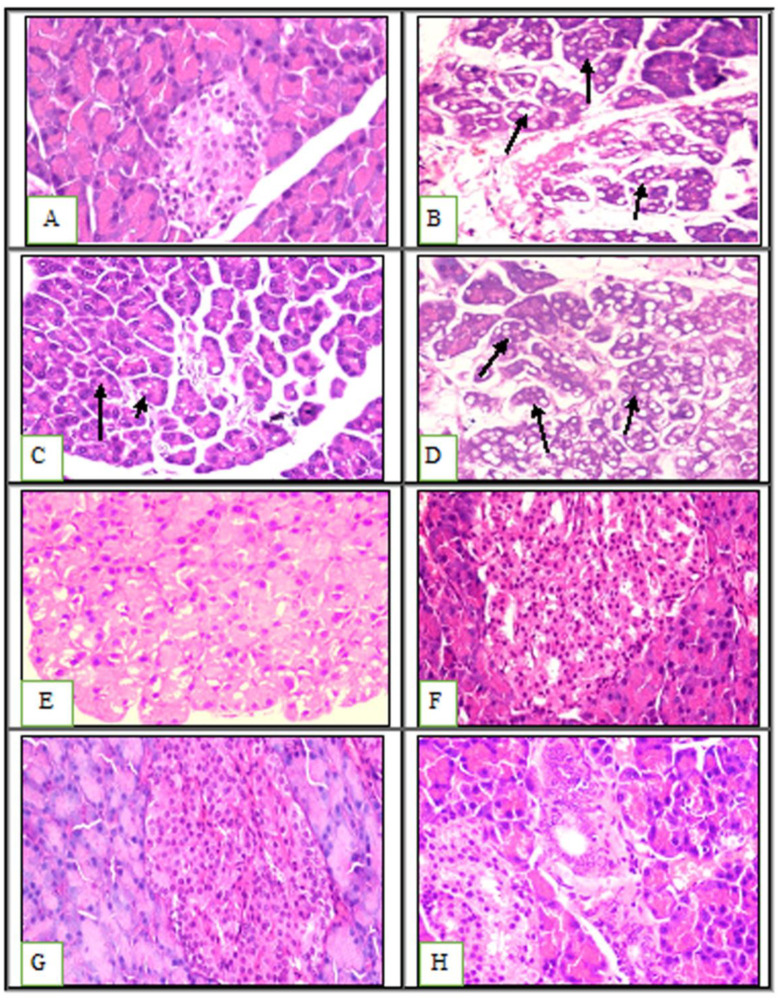
Histopathological examination of the pancreatic tissue. (**A**) Normal pancreatic tissue (H&E ×400). (**B**) Massively vacuolated acinar epithelium (arrows) (H&E ×400). (**C**,**D**) Partially vacuolated acinar epithelium (arrows). (**E**,**F**) Normal pancreatic tissue (H&E ×400). (**A**) Negative control. (**B**) Positive control. (**C**,**D**) Green tea. (**E**) Chia seed. (**F**) Chitosan. (**G**) Probiotic. (**H**) Mixture. A 50 µM bar scale was used for all the tissues.

Upon microscopic examination, the pancreatic tissues of the negative control group revealed normal histological features in both the exocrine and endocrine tissues. The islet of Langerhans showed normal cellular arrangement and an evaluation of the acinar structure revealed the islet of Langerhans’ normal proteinaceous eosinophilic materials (Figure 5A). The positive control group showed atrophied pancreatic lobules and vacuolated acinar epithelial lining (Figure 5B). 

The green tea-treated animals showed vacuolation only in some acinar epithelial linings compared with the positive control group, which showed massive vacuolation of the acinar tubular epithelial lining and marked interlobular edema (Figure 5C,D). Moreover, the animals treated with chia seeds, chitosan, probiotics, and their mixture displayed normal pancreatic parenchyma (Figure 5E–H). Lutgendorff et al. previously described the beneficial effects of probiotics in treating acute pancreatitis and proposed that probiotics enhance glutathione biosynthesis, which ameliorates inflammation and acinar cell injury [54].

### 2.4. Molecular Docking Studies

Green tea is rich in catechins, which majorly contribute to its antioxidant benefits. Moreover, multiple studies have shown that it lowers blood cholesterol levels, prevents hypertension, and demonstrates several pharmacological benefits including anti-inflammatory activity [55,56]. Phenolics are the primary bioactive components behind green tea’s medicinal benefits, and most of these compounds are catechin derivatives, such as (+)-catechin, (-)-epicatechin gallate, (+)-gallocatechin, and epigallocatechin gallate [57,58]. Chia seeds are excellent food supplements as they can potentially protect against free radicals [59]. Polyphenol compounds such as gallic, caffeic, chlorogenic, epicatechin, daidzein, and genistein were found in chia seeds [60,61]. The antioxidant potential of natural phenols may protect against certain conditions including atherosclerosis, diabetes, stroke, and neurological disorders [62,63].

Similar to NSAIDs, phenolic substances suppress the activity of pro-inflammatory mediators, except for COX. Additionally, several phenolics can regulate inflammatory and oxidative signaling pathway mediators such as nuclear factor-kappaB (NF-κB) [57].

We performed molecular docking to better understand the probable binding, affinity, and binding pattern of the major bioactive constituents present in chia seeds and green tea with β-hydroxy β-methylglutaryl-CoA (HMGCoA) reductase, xanthine oxidase, and COX-2 enzymes. For these studies, we selected nine of the most common phenolic compounds found in chia seeds and green tea including gallic acid, caffeic acid, chlorogenic acid, daidzin 7-o-glucoside, genistein, (+)-catechin, (-)-epicatechin gallate, (+)-gallocatechin, and epigallocatechin gallate. Version 2019.02 of the MOE was used to analyze all the docking steps. The calculated RMSD between the co-crystallized and docked poses was 0.75 Å for oxypurinol, 0.85 Å for LVA, and 0.55 Å for D75, which validated the applied docking. The docking scores and RMSD are shown in Table 3.

#### 2.4.1. Docking of Compounds into Xanthine Oxidase (XO) Binding Site 

**Figure 6:**
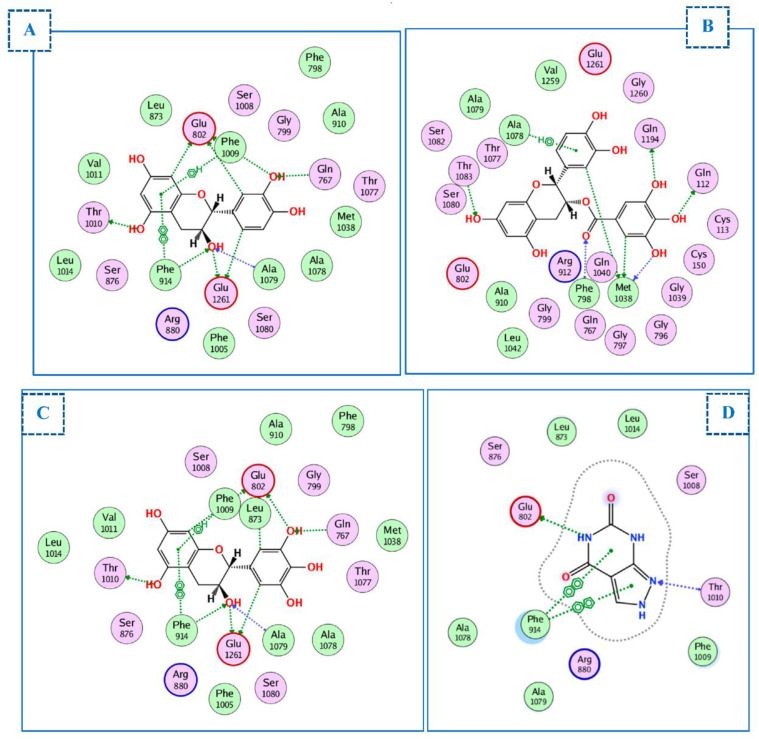
Two-dimensional diagram of (**A**) (+)-catechin, (**B**) (-)-epicatechin gallate, (**C**) (+)-gallocatechin, and (**D**) oxypurinol into the binding site of xanthine oxidase (PDBID: 7dnv).

To comprehend the binding mode of the nine compounds, we performed molecular docking studies using xanthine oxidoreductase (XO) bound to oxypurinol (PDB ID: 7dnv). The RMSD between the co-crystallized and docked poses of oxypurinol was 0.75 Å; furthermore, the docking score was −21.3 kcal/mol. All compounds docked correctly into the active binding site of oxypurinol (Table 3) with docking scores ranging from −14.7 to −24.3 kcal/mol. The highest docking scores were −22.4 kcal/mol for (+)-catechin, −21.7 kcal/mol for (-)-epicatechin gallate, and −24.3 kcal/mol for (+)-gallocatechin. For these three compounds, the restricted access channel of the XO binding site was occupied with Phe914, Phe1009, Phe1013, Val1011, Leu649, and Leu1014. (+)-catechin appeared to significantly participate in the inhibitory activity as it could form hydrophobic interactions with Phe914 and Phe1009 and nine hydrogen bonds (H-bonds) with Glu802, Glu1261, Gln767, Ala1079, Phe914, and Thr1010, compared with oxypurinol, which forms only two H-bonds with Glu802 and Thr1010 and two arene–arene interactions with Phe914. (-)-epicatechin gallate binds to the active site via H-bonding with Thr1083, Gln1194, Gln112, Met1038, and Phe798 in the narrow channel. (+)-gallocatechin formed two hydrophobic interactions with Phe914 and 1009 and nine H-bonds with Glu802, Gln767, Ala1079, Glu1261, and Phe914 (all essential amino acids (Figure 6A–D).

#### 2.4.2. Docking of Compounds into the HMG-CoA Reductase Binding Site

**Figure 7:**
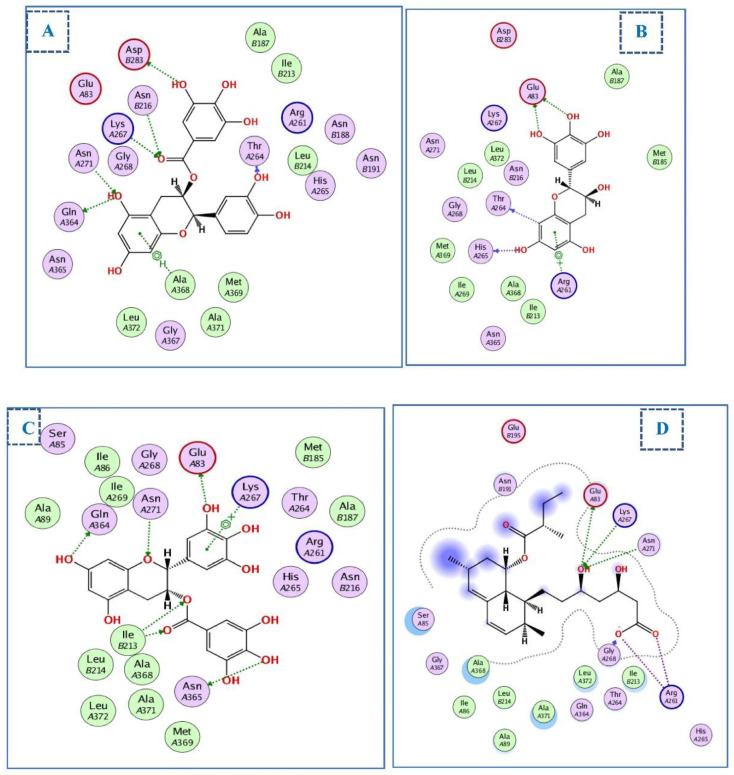
Two-dimensional diagram of (**A**) (-)-epicatechin gallate, (**B**) (+)-gallocatechin, (**C**) epigallocatechin, and (**D**) LVA into the binding site of HMG-CoA reductase (PDBID: 1t02).

The structural coordinates of the HMG-CoA reductase were determined using the PDB ID:1t02 [64]. Molecular docking demonstrated that the main interactions between the nine chosen compounds and HMG-CoA reductase resembled those of its co-crystal ligands, lovastatin (LVA). As shown in Figure 6, LVA engages in many crucial interactions including those with Lys267, Asn271, Ala368, Glu83, and Arg261. The most significant compounds were (-)-epicatechin gallate, (+)-gallocatechin, and epigallocatechin, with docking scores of −17.7, −14.4, and −14.5 kcal/ mol, respectively, comparable to the LVA docking value (−14.4 kcal/mol) (Table 3).

(-)-epicatechin gallate forms an arene–H bond with Ala368, which is located on the hydrophobic face of an α-helix within the main domain required for HMG-CoA binding. Additionally, the hydroxyl group at position 5 of the chromanyl moiety was engaged in two H-bonds with Asn71 and Gln 364. Moreover, the carbonyl group acts as an acceptor of hydrogen bonds with Asn216 and Lys267. Furthermore, the trihydroxy benzoate moiety forms an H-bond with Asp283. Similarly, (+)-gallocatechin and epigallocatechin gallate fit perfectly within the active site of HMG-CoA. The trihydroxy benzoate moiety may be responsible for the superior binding of (-)-epicatechin gallate and epigallocatechin gallate over (+)-gallocatechin. (+)-gallocatechin forms two H-bonds with the catalytic amino acid, Glu-83 (Figure 7A–D).

#### 2.4.3. Docking of Compounds into the COX-2 Binding Site

**Figure 8:**
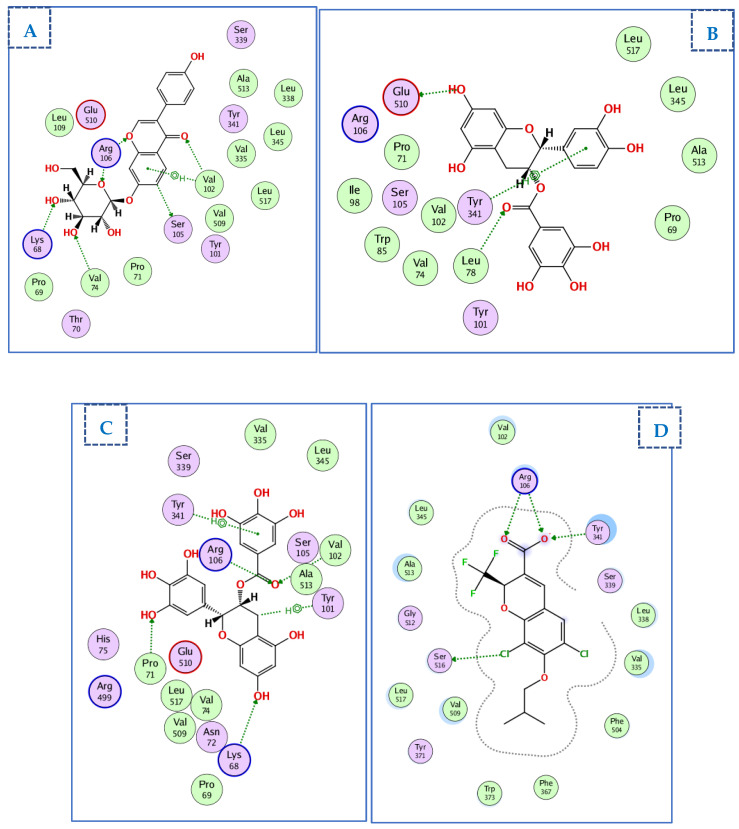
Two-dimensional diagram of (**A**) daidzin7-O-glucoside, (**B**) (-)-epicatechin, (**C**) epigallocatechin, and (**D**) D72 moieties in the binding site of COX-2 (PDBID: 3ntg).

The crystal structure of COX-2 with its selective inhibitor (D72) was obtained using PDB ID 3ntg [65]. D72 exhibited one ionic bond interaction with Arg106 via its carboxylate ion, two H-bonds with Arg106 and Try341, and an H-bond between one chlorine atom and Ser516. Selected compounds were docked onto the same binding site on COX-2 with docking scores ranging from −10.6 to −21.5 kcal/mol (Table 3). Figure 8 shows the compounds with the highest docking values and the ligand D72.

(-)-epicatechin gallate displayed the highest docking score energy (−21.5 kcal/mol), forming a hydrophobic interaction with Try341and two H-bonds with Glu510 and Leu78. Daidzin7-O-glucoside showed 6 H-bonds and an arene–H bond with three H-bonds between the glucose moiety and Arg106, Val74, and Lys68, and three H-bonds between the chromanyl moiety and Arg106, Val102, and Ser105. Conversely, the two 3,4,5 trihydroxy phenyl groups in epigallocatechin gallate were important for binding with COX-2, forming H-bonding interactions with Val102, Arg106, Lys68, and Pro71, and two hydrophobic interactions with Try101 and Try341 (Figure 8A–D).

In summary, all nine compounds displayed strong interactions with their target enzymes, which allowed us to determine the roles of chia seeds and green tea in preventing obesity. Overall, (-)-epicatechin gallate showed the best docking results and was able to bind strongly to all three enzymes through a variety of interactions.

## 3. Materials and Methods

### 3.1. Materials

#### 3.1.1. Procurement, Preparation, Purification, and Activation of the Probiotics (*L. plantarum*) 

Firstly, *L. plantarum* (ATCC 14917, Lp 39 [IAM 12477]) cultures were subcultured in MRS broth (Acumedia, Lansing, MI, USA). Then, they were streaked on MRS agar (Acumedia, Lansing, MI, USA) to check the purity and obtain a single colony, which was subsequently subcultured by inoculating it in 50 mL of MRS broth and incubating at 37 °C for approximately 24–48 h in a gas incubator in a microaerophilic state [66]. *L. plantarum* was re-suspended in sterile phosphate-buffered saline (PBS) until it reached 5 × 10^8^ CFU/mL [67]. The bacteria were freshly prepared daily during the eight week-long experimental period and their concentrations were determined using the McFarland method [68]. The standard and test suspension were prepared using new and pure culture to test the organisms and inoculate in MRS broth.

#### 3.1.2. Procurement and Preparation of the Prebiotics 

The green tea leaves and chia seeds were purchased at an Upper Egypt local market. The green tea leaf extract was obtained using 80% ethanol after macerating the leaves for 24 h [69]. The solution was lyophilized after filtering, and the prepared powder was dissolved in distilled water before feeding the rats. One gram of chitosan (Sigma-Aldrich, Gillingham, UK) was homogenized after being dissolved for six hours in 40 mL of a two-weight percent acetic acid in distilled water solution [70].

### 3.2. Methods

#### 3.2.1. Animal Models

Before the adaptation test, forty-eight adult male Sprague–Dawley rats (age: 7 weeks; body weight: 100–110 g) were subjected to perception for seven days. The animals were retained in stainless steel cages and the room temperature was maintained at approximately 24 ± 2 °C with a 12-hour light/12-hour dark cycle. The rats received treatment with two types of diet: (1) the standard basal diet (SBD), and (2) the high-fat diet (HFD), which consisted of altered SBD to expand the fat content while diminishing the polysaccharide amount. Table 4 lists the ingredients in both diets. All the animals were placed on an ordinary feeding regimen for one week with free access to water during the experiment. The experimental animals were subsequently split into seven groups, each with eight rats, as shown in Table 5. The study received approval from Sadat City University’s ethical committee for the care and use of animals in education and scientific research (Approval No. 12-214 at 21/6/2020).

#### 3.2.2. Sampling and Evaluation of Biological Parameters

See supplementary section for further details [71,72]. 

#### 3.2.3. Biochemical Analysis Tests

See supplementary section for further details [73,74,75,76,77,78,79,80,81,82,83]. 

#### 3.2.4. Histological Examination

See supplementary section for further details.

#### 3.2.5. Statistical Analysis

See supplementary section for further details.

#### 3.2.6. Docking Studies

**Figure 9:**
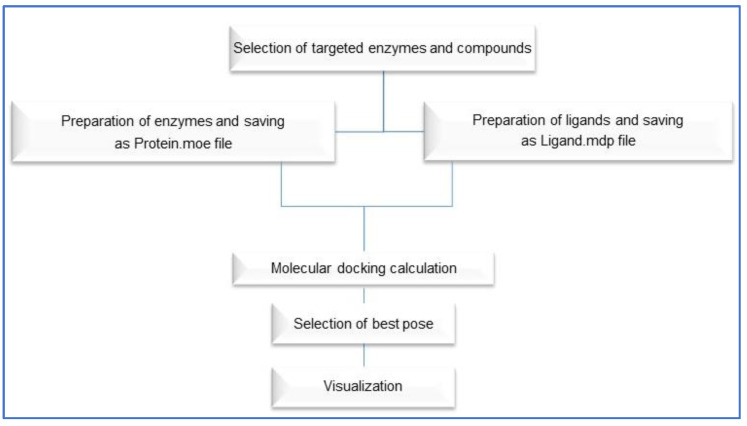
Flow chart of docking steps using MOE 2019.

The X-ray structures of HMGCoA reductase, xanthine oxidase, and COX-2 enzymes were downloaded from the Protein Data Bank (PDB) using the following IDs: 1t02, 7dnv and 3ntg, respectively. The entire docking was performed using a molecular operating environment (MOE) 2019, Cozza, and Moro, 2009 [83], starting with the three enzymes and nine compounds including the major components of chia seeds and green tea using the default parameters. The active site of each target was determined from the binding of the corresponding co-crystalized ligand. To validate the applied docking approach, each co-crystalized ligand was re-docked onto its targets and active site, and the resulting RMSD was calculated for the docked positions [84]. Additionally, the docking scores generated in the previous steps for the co-crystalized ligands were used as comparative benchmark values for the nine compounds. Finally, the docking results for the nine compounds in the active sites of the three enzymes were conducted using the validated docking parameters. We generated the 2D interaction diagrams between the docked ligands and their potential targets using a MOE. The docking steps are summarized in Figure 9.

## 4. Conclusions

Obesity is one of the most serious metabolic diseases worldwide that affects both developed and developing countries. A diet enriched with probiotics and prebiotics, or food ingredients could be a good alternative approach for controlling obesity given that anti-obesity medications are very expensive and have unwanted side effects; *Lactiplantibacillus plantarum* combines with chia seeds, green tea, and chitosan in alleviating hyper-lipidemia and inflammation. Our results showed a synbiotic effect of this combination in managing obesity and its related problems, especially oxidative stress and/or organ damage. It decreased serum catalase, superoxide dismutase, and liver malondialdehyde levels. It also reduced body fat accumulation and prevented liver damage in obese mice fed a HFD. A histopathological examination revealed a reduction in obesity-related injuries in the liver, kidney, and pancreas. Molecular docking studies were conducted that presented a potential role of the chia seeds, green tea, and chitosan as inhibitors for xanthine oxidase, HMG-CoA reductase, and COX-2, especially. (-)-epicatechin gallate, probiotics, and prebiotics could be a novel approach for the obese population to reduce obesity. However, further clinical studies on obesity are required.

## Figures and Tables

**Table 1:** Grading of the hepatic lesions.

Grade	Grade Description
0	No apparent injury by light microscopy
I	Hepatocytes swelling
II	Hepatocytes ballooning
III	Lipid droplets in hepatocytes

**Table 2:** Grading of the renal scoring.

Grade	Grade Description
0	Normal histology
I	Tubular epithelial cell degeneration without significant necrosis or apoptosis
2	Tubular epithelial cell necrosis and apoptosis are less than 25%

**Table 3:** Binding energies (kcal/mol) obtained after docking.

No.	Compound Name	Docking Scores kcal/mol (RMSD Å)		
		Xanthine oxidase	HMG-CoA	COX-2
1	Gallic Acid	−16.8 (1.01)	−10.6 (0.88)	−11.1 (1.15)
2	Caffeic Acid	−17.6 (0.52)	−9.8 (0.89)	−10.8 (1.19)
3	Chlorogenic Acid	−20.2 (0.88)	−13.5 (1.15)	−14.2 (1.03)
4	Daidzin7-O-Glucoside	−17.2 (1.24)	−11.1 (1.12)	−15.0 (0.95)
5	Genistein	−14.7 (0.86)	−10.9 (0.65)	−12.7 (0.75)
6	(+)-Catechin	−22.4 (0.44)	−12.8 (0.89)	−10.6 (0.72)
7	(-)-Epicatechin Gallate	−21.5 (1.04)	−17.7 (1.03)	−21.5 (1.01)
8	(+)-Gallocatechin	−24.3 (1.06)	−14.4 (1.24)	−14.5 (0.82)
9	Epigallocatechin Gallate	−16.9 (0.95)	−14.5 (0.99)	−16.9 (1.06)
11	Oxypurinol	−21.3	-	-
12	LVA	-	−14.4	-
13	D72	-	-	−15.0

**Table 4:** Chemical composition of the SBD and HFD.

Constituent	(%) in SBD	(%) in HFD
Corn starch	56.1	26.1
Casein	14	14
Sucrose	10	10
Corn oil	10	10
Cellulose	5	5
Minerals	3.5	3.5
Vitamins	1	1
Methionine	0.18	0.18
Choline chloride	0.25	0.25
Tert-butyl hydroquinone	0	0
Lard	0	30

SBD: standard basal diet, HFD: high-fat diet.

**Table 5:** Group distribution and experimental design.

Intake Type	Group						
	1	2	3	4	5	6	7
	SBD	HFD	HFD	HFD	HFD	HFD	HFD
Chia seed (%) mg/kg diet	–	–	20	–	–		20
Chitosan (mg/kg diet)	–	–	–	400	–		400
Green tea extract (mg/kg diet)	–	–	–	–	200		200
*L. plantarum* (CFU)	–	–	–	–	–	1 × 10^6^	1 × 10^6^

SBD: standard basal diet, HFD: high-fat diet, CFU: colony-forming unit.

## Data Availability

All data generated or analyzed during this study are included in the published article.

## Supplementary Materials

The following supporting information can be downloaded at: https://www.mdpi.com/article/10.3390/ijms232012200/s1.
