# Supplementary material for: In Vivo and In Silico Investigation of the Anti-Obesity Effects of Lactiplantibacillus plantarum Combined with Chia Seeds, Green Tea, and Chitosan in Alleviating Hyperlipidemia and Inflammation
Source: Int J Mol Sci. 2022 Oct 13;23(20):12200. doi: 10.3390/ijms232012200 (PMC9602495; doi:10.3390/ijms232012200)
Supplement: Supplementary file 1 [file ijms-23-12200-s001.zip › ijms-1900597-supplementary.pdf]

Results

3.1 Effect of prebiotic and prebiotic on the weight of HFD rats

	Groups						
	control positive	control negative	Green tea	Chia seed	chitosan	Probiotics	Mixture
**BWG (g/6 weeks)**	$113\pm14_{a,c,d}$	$62\pm6_{a,b}$	$125\pm13_{a,c,d}$	$97\pm6_{a,c,d}$	$95\pm5_{a,c,d}$	$113\pm2_{c}$	$77\pm4_{b,d}$
**Relative liver weight (g/100 g BW)**	$.042712236987819\pm0.000559994481379_{a}$	$.029219619683247\pm0.000218483174503_{b}$	$.034089271089272\pm0.000409025933145_{c}$	$.030324235385211\pm0.000765483391039_{b,c}$	$.028642592592593\pm0.000765675240156_{b,c}$	$.028712527912528\pm0.001274697693556_{b,c}$	$.032883153322178\pm0.001405983246608_{a,b,c}$
**Relative kidney weight (g/100 g BW)**	$.016923920265781\pm0.00109889034826_{a}$	$.007958620689655\pm0.000196198513042_{b}$	$.006403767403768\pm0.000219927264533_{b}$	$.006626945412312\pm0.000334652095537_{b}$	$.006907407407408\pm0.000271585296708_{b}$	$.006670147070148\pm0.000806872127977_{b}$	$.007156973108193\pm0.000538917132911_{b}$
**Relative internal fats weight (g/100 g BW)**	$.014060132890366\pm0.000810278116908_{a}$	$.008004873139468\pm0.001181823969081_{a}$	$.011229320229321\pm0.001572919378379_{a}$	$.011051555039360\pm0.00109751238394_{a}$	$.018935185185185\pm0.001514539728713_{a}$	$.007613490413491\pm0.00033142483449_{a}$	$.007218509316071\pm0.000871346047874_{a}$
**Relative subcutanious weight (g/100 g BW)**	$.015749612403101\pm0.000202456223229_{a}$	$.009262693998623\pm0.000533501050393_{b}$	$.015543407043408\pm0.00148700864881_{a,b}$	$.021449993547555\pm0.001053651368442_{a}$	$.020164814814815\pm0.00266797871529_{a,b}$	$.011167813967814\pm0.000375126965643_{b}$	$.010859914698941\pm0.00035877601639_{b}$

Note: Values in the same row and subtable not sharing the same subscript are significantly different at p< .05 in the two-sided test of equality for column means. Cells with no subscript are not included in the test. Tests assume equal variances.[1]

1. Tests are adjusted for all pairwise comparisons within a row of each innermost subtable using the Bonferroni correction.

Comparisons of Column Means[a]

	Groups						
	control positive (A)	control negative (B)	Green tea (C)	Chia seed (D)	chitosan (E)	Probiotics (F)	Mixture (G)
BWG (g/6 weeks)						B(.032)<br>G(.041)	

3.2 Effect of prebiotic and prebiotic on the lipid profile

	groups/test						
	**control positive**	**control negative**	**Green tea**	**chia seed**	**chitosan**	**Probiotic**	**Mixture**
**HDL**	$38\pm1_{a}$	$50\pm1_{b}$	$40\pm2_{a}$	$41\pm3_{a}$	$41\pm1_{a}$	$43\pm1_{a,b}$	$42\pm1_{a}$
**T.G**	$187\pm5_{a}$	$75\pm4_{b}$	$144\pm5_{c,d}$	$166\pm9_{a,c}$	$166\pm5_{a,c}$	$164\pm2_{a,c}$	$126\pm2_{d}$
**TC**	$213\pm4_{a}$	$112\pm5_{b}$	$188\pm5_{c,d}$	$165\pm6_{c}$	$183\pm5_{c}$	$166\pm2_{c}$	$121\pm4_{b}$
**VLDL**	$36.7\pm1.3_{a}$	$14.9\pm0.8_{b}$	$28.8\pm1.1_{c,d}$	$33.3\pm1.7_{a,c}$	$33.2\pm0.9_{a,c}$	$32.8\pm0.3_{a,c}$	$25.2\pm0.4_{d}$
**LDL**	$135.9\pm3.4_{a,e}$	$58.1\pm3.4_{b}$	$122.5\pm5_{a,d,e}$	$90.7\pm9.6_{c}$	$110.5\pm5.1_{a,c}$	$96.2\pm1.1_{c,d}$	$58.5\pm5.3_{b}$

Note: Values in the same row and subtable not sharing the same subscript are significantly different at p< .05 in the two-sided test of equality for column means. Cells with no subscript are not included in the test. Tests assume equal variances.[1]

1. Tests are adjusted for all pairwise comparisons within a row of each innermost subtable using the Bonferroni correction.

Comparisons of Column Means[a]

	groups/test						
	control positive (A)	control negative (B)	Green tea (C)	chia seed (D)	chitosan (E)	probiotic (F)	Mixture (G)
HDL		A(.021)					
T.G	B(.001)<br>G(.007)		B(.014)	B(.018)	B(.004)<br>G(.042)	B(.001)<br>G(.003)	B(.010)
TC	B(.002)<br>F(.009)<br>G(.002)		B(.009)<br>G(.009)		B(.012)<br>G(.012)	B(.013)<br>G(.011)	
VLDL	B(.004)<br>G(.033)		B(.014)	B(.018)	B(.004)<br>G(.042)	B(.001)<br>G(.003)	B(.010)
LDL	B(.002)<br>F(.011)<br>G(.007)		B(.012)<br>G(.026)		B(.029)	B(.012)	

Results are based on two-sided tests assuming equal variances. For each significant pair, the key of the smaller category appears in the category with the larger mean.

Significance level for upper case letters (A, B, C): .05

a. Tests are adjusted for all pairwise comparisons within a row of each innermost subtable using the Bonferroni correction.

3. Materials and Methods

3.2. methods

3.2.2. Sampling and Evaluation of Biological Parameters

After the sixth week, the rats were abstained from food for 12 h and the animals were anesthetized and blood samples were collected from the orbital sinus [71] and kept in dry test tubes then sacrificed by decapitation under thiopental anesthesia (50 mg/kg) [72] by well-trained personnel according to AVMA Guidelines for the Euthanasia of Animals: 2020 Edition. All the samples were centrifuged at 1381 rpm for 15 min to obtain the serum, which was stored at 20 °C for further biochemical analysis. The rats were dissected immediately and after surgically removing the pancreas, kidney, and liver, the organs were washed in normal saline (NaCl, 0.9%), dried using Whatman papers, and weighed immediately. The body weights (BW) of the test rats were recorded at the beginning and end of the experiment to calculate the body weight gain (BWG). Additionally, relative weights of the kidney and liver of all the test groups were estimated.

3.2.3. Biochemical Analysis Tests

Serum creatinine, urea and uric acid were determined by the colorimetric method [73]. Serum aspartate aminotransferase (AST), alanine aminotransferase (ALT), alkaline phosphatase (ALP), albumin and total protein concentrations were determined calorimetrically [74,75].

Serum total lipids (TL), cholesterol (TC), triglycerides (TG) and high-density lipoprotein cholesterol (HDL-C) content were analyzed by the enzymatic colorimetric method [73,76,77], while lowdensity lipoprotein cholesterol (LDL-C) and very low-density lipoprotein cholesterol (VLDL-C) were calculated according to the equation of Friedewald et al. [78].

Friedewald's equation:

$$LDL\ (mg/dL) = TC - [HDL + TG/5].$$

$$VLDL = TG/5$$

$$Risk\ 1 = TC/HDL \quad Risk\ 2 = LDL/HDL$$

Livers and kidney were removed, freed from adhering tissues, and washed with ice-cold normal saline solution (0.9%). The weight of all the organs was taken only after drying the tissue. One g tissue was homogenized in 10 mL of 0.2 M tris-HCl with the help of homogenizer. The homogenate was centrifuged at 10,000 rpm for 20 min at 4 °C. The supernatant obtained was used for the estimation of superoxide dismutase (SOD) [79,80], malondialdehyde (MDA) [81] and catalase (CAT) [82].

3.2.4. Histological Examination

The liver, kidney, and pancreas tissues were fixed in 10% neutral buffered formalin. They were then cut, washed, and dehydrated using increasing amounts of alcohol, cleared in xylene, paraffin-embedded, sectioned at 4–6 μm thickness, and stained by hematoxylin and eosin for microscopic examination.

3.2.5. Statistical Analysis

The statistical data were analyzed using the IBM Statistical Package for the Social Sciences (SPSS) version 25. The laboratory parameters were expressed as mean and standard deviation (SD) for continuous data. A post hoc Bonferroni correction test was conducted to show similarities among the examined parameters in all groups, implementing 2-way ANOVA. p-value was considered significant when < 0.05.
